# Moving on in hydrocephalus imaging: from 2D to 3D biomarkers

**DOI:** 10.1007/s00701-026-06985-2

**Published:** 2026-07-21

**Authors:** Raffaele Da Mutten, Rafael Turczynski Holmgren, Erik Edström, Adrian Elmi-Terander, Victor Egon Staartjes

**Affiliations:** 1 Machine Intelligence in Clinical Neuroscience and Microsurgical Neuroanatomy (MICN) Laboratory, Department of Neurosurgery, Clinical Neuroscience Center, University Hospital Zurich, University of Zurich, Zurich, Switzerland; 2 Department of Neurosurgery, Cantonal Hospital of Lucerne, Lucerne, Switzerland; 3 Department of Neurosurgery, Linköping University, Linköping, Sweden; 4 Department of Biomedical and Clinical Sciences, Linköping University, Linköping, Sweden; 5 Department of Clinical Neuroscience, Karolinska Institute, Nobels Väg 6, Solna, 171 77 Stockholm, Sweden; 6 Capio Spine Center Stockholm, Löwenströmska Hospital, Stockholm, Sweden

**Keywords:** Hydrocephalus, Segmentation, Volumetry, 3D, Biomarkers, Imaging

## Abstract

**Purpose:**

Linear two-dimensional indices such as the Evans index, callosal angle, and fronto-occipital horn ratio remain the clinical standard for hydrocephalus assessment, yet are limited by measurement variability, insensitivity to spatial CSF redistribution, and reduced sensitivity to volumetric change over time. This narrative review aims to summarize established two-dimensional indices, their structural limitations, and describe how automated segmentation and radiomic feature extraction enable three-dimensional assessment across four clinical domains.

**Methods:**

A narrative literature review was conducted using PubMed. Studies addressing hydrocephalus imaging biomarkers, ventricular volumetry, automated segmentation, radiomics, and machine learning applications were reviewed. Reference lists of relevant articles were hand-searched for additional sources. The literature was synthesized across four clinical domains: pediatric hydrocephalus monitoring, differential diagnosis of ventriculomegaly, preoperative prediction of response to cerebrospinal fluid diversion, and longitudinal post-treatment follow-up.

**Results:**

Across pediatric hydrocephalus monitoring, differential diagnosis of ventriculomegaly, preoperative prediction of response to CSF diversion, and post-shunt longitudinal follow-up, three-dimensional volumetric and radiomic approaches consistently outperform linear indices. Machine learning models report AUCs exceeding 0.9 for differential diagnosis and shunt response prediction. Automated segmentation has reached excellent performance for detection tasks, and volumetry is more sensitive to postoperative change than the Evans index.

**Conclusions:**

Despite strong metric performance, clinical translation remains limited by small single-centre datasets, missing external validation, and undefined thresholds for clinically meaningful volumetric change. Embedding validated tools into radiological workflows and clinical guidelines will be essential before three-dimensional biomarkers can improve routine hydrocephalus care.

## Introduction

For decades, the quantitative assessment of hydrocephalus has rested on a handful of linear measurements such as the Evans’ index (EI), the callosal angle (CA), or the frontal and occipital horn ratio (FOHR) [16, 36, 64]. Their enduring presence in clinical practice and guidelines stems from their simplicity: a single ratio or angle, derivable for any standard scan [59, 73]. Yet, at times, hydrocephalus, especially idiopathic normal pressure hydrocephalus (iNPH), presents a diagnostic challenge: clinical symptoms are often subtle and non-specific, overlapping with normal aging and neurodegeneration [23, 68, 73]. Therefore, imaging is often consulted as an objective measure, but radiological signs themselves can be equally indeterminate, with changes that are small, gradual, and difficult to distinguish from atrophy without precise quantification [5, 68, 92]. Linear measurements compound this problem, as they simplify a complex dynamic three-dimensional structure into a single numerical value, thereby overlooking spatial positioning, local phenomena, and temporal factors. As a consequence, research over the past decades has explored more complex variables to better understand this complex pathology. After volumetry alone, morphometric features, analyzing shape and local distributions, were introduced. In recent years, these were combined and extended using machine learning. As their diagnostic and prognostic performance clearly outperforms simple linear measurements, this narrative review aims to summarize how hydrocephalus has been evaluated historically, explore new approaches that have shown promise, and discuss potential future directions for research in this field.

## The era of 2D biomarkers: strengths and structural constraints

### Classical two-dimensional indices

First introduced in 1942 on pneumoencephalograms, the EI divides the transverse diameter of the anterior horns by the maximum diameter of the skull [16, 17]. A value above 0.3 is considered indicative of hydrocephalus. It has been the dominant radiological indicator of ventriculomegaly for many decades. It is generally appreciated for its ease of use. Its diagnostic performance is reasonable, characterized by a high sensitivity and low specificity [68]. As it is usually not adapted for age and gender, up to 29% of healthy controls also have an Evans index of above 0.3, illustrating the low specificity [5]. While interrater agreement is generally high, slice angulation and selection are inconsistent, introducing problems with reproducibility [68, 92].

Similarly, the CA is obtained on a standard coronal plane at the level of the posterior commissure [36]. A large value is generally interpreted as suggestive of atrophy as a cause of ventriculomegaly, whereas a small number indicates iNPH [36]. In contrast to the aforementioned EI the CA has higher specificity [36, 68]. Slice angulation is a confounder as well, with significant reductions in inter-rater agreement with only small rotational errors [51]. Complementary, the subarachnoid CSF space can be assessed more qualitatively through the DESH (disproportionately enlarged subarachnoid space hydrocephalus) patterns—defined by enlarged Sylvian fissures and tight high-convexity sulci [59, 91]. More generally, the brain per ventricle ratio (BVR) or ventricle to brain ratio (VBR) assess the quotient of ventricular volume to brain volume. The BVR has been shown to change with higher sensitivity after shunt surgery than EI [99].

Initially defined in pediatric patients, the FOHR can also be evaluated on sonography. The maximum diameter of the frontal and occipital horns is added and then divided by twice the biparietal diameter [64]. Furthermore, the cella media ratio compares the diameter of the tabula externa of the skull with the extension of the bodies of lateral ventricle at the cella media [65].

### Strengths

Although their diagnostic performance is limited, 2D-measures are appreciated for multiple reasons [68]. First, these conventional indices are used due to their speed and ease of use. They also reflect – to some extent and especially in pronounced cases – what an experienced rater sees at first glance; the relative distribution of CSF-space and partial abnormalities specific for pathologies. Second, they require minimal to no post-processing of the scans, which further facilitates their application. Third, they are well integrated into clinical workflows and guidelines. Fourth, when multiple characteristics are combined, e.g. into the iNPH Radscale [45], the combined diagnostic value increases [46]. Last, they can be used as screening tools for further inspection and analysis.

### Intrinsic limitations

**Fig. 1:**
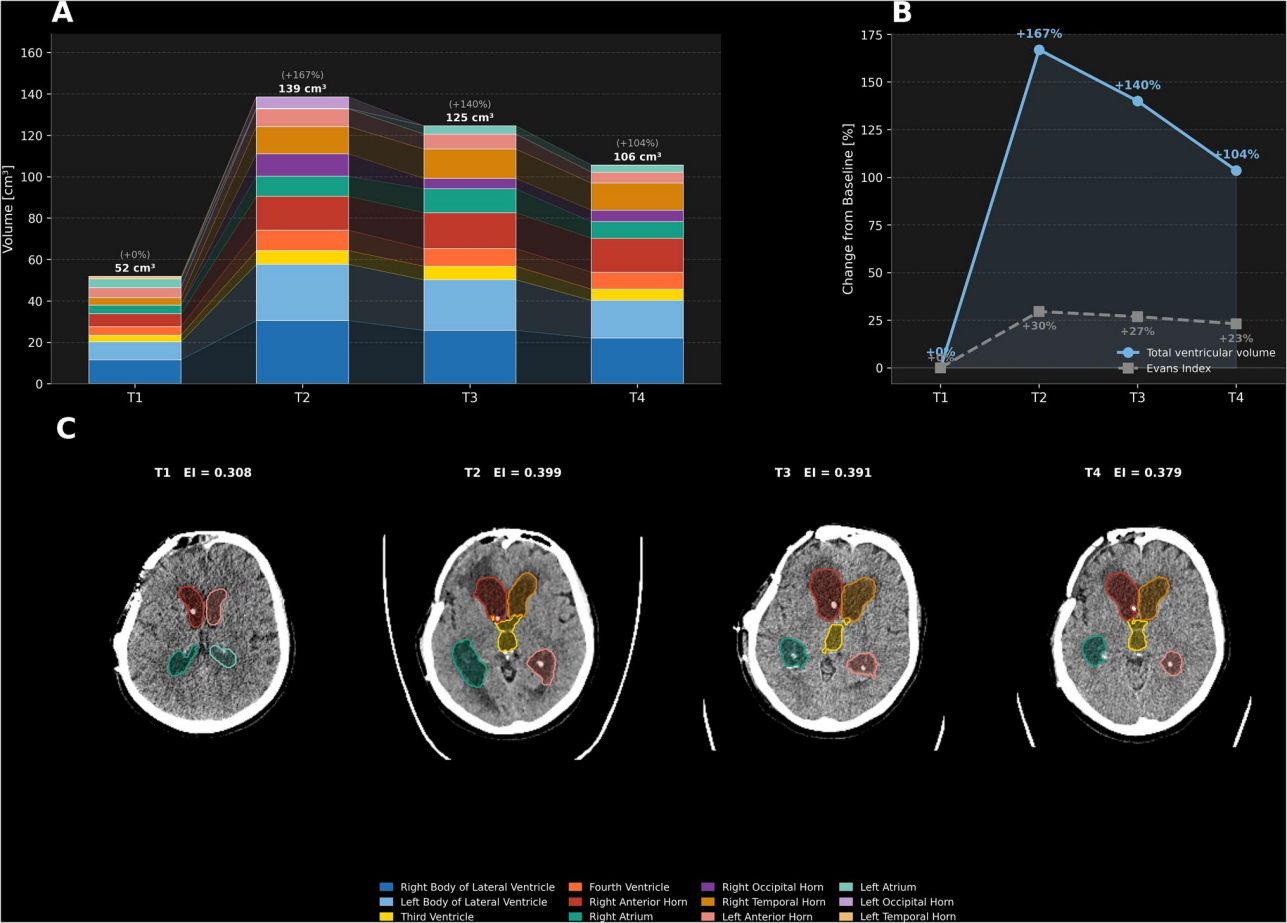
Illustrative case of changes in ventricular width and volume. The patient suffered from an aneurysmal subarachnoid hemorrhage, requiring a VP-shunt. After a normal postoperative control (timepoint 1, T1), she was readmitted four years later with impaired gait and confusion. CT control (timepoint 2, T2) showed increased ventricular width. Shunt-dysfunction was identified, and the VP-shunt was replaced. Postoperatively, the ventricular width decreased again in the direct postoperative control (timepoint 3, T3) and at follow-up (timepoint 4, T4), where symptoms also weakened. Masks were automatically generated and required minimal manual adjustment. **A** Automatically extracted volumes per subregions at four different timepoints, as well as the percentage-wise change of the entire volume compared to the first timepoint. Twelve anatomical subregions were considered. **B** Comparison of the percentage-wise change of total ventricular volume and Evans index compared to baseline. The ventricular volume changed to a larger magnitude than the Evans index, illustrating the improved sensitivity of ventricular volume compared to linear indices. **C** Axial CT slices from the four time points with automatically created masks as an overlay. Manually measured and calculated Evans index is also reported. T1: Baseline after initial VP-shunt placement. T2: Readmission with symptomatic shunt dysfunction. T3: Postoperative condition after revision surgery. T4: Follow-up

Despite their practical advantages, these well-established metrics are increasingly being evaluated critically due to their inherent limitations. Two-dimensional assessment requires the selection of a slice. Especially in CT, where head positioning is not rigidly standardized, variations in plane angulation introduce significant measurement heterogeneity [92]. Moreover, linear metrics reduce complex three-dimensional CSF-distribution patterns to isolated aspects, e.g., only reflecting frontal horn extension while neglecting the temporal horns or convexity subarachnoid spaces. Further, ventricular volume (VV) is more sensitive to changes over time than linear measurements [15, 61]. This is illustrated by a selected case in Fig. 1. Clinical improvement after shunt surgery has been shown to correlate better with volumetry than the EI, as shown by Neikter et al. [61], and VV reduction occurs more strongly along the cranio-caudal z-axis , which is insufficiently represented by the standard linear features. This also highlights the complexity of spatial expansion in CSF-pathologies, which is hard to grasp in a single slice. To address this limitation, the concept of the Evans index has been applied to the z-axis, named the z-Evans index [100]. Furthermore, volumetry has emerged as an alternative way of evaluating hydrocephalus.

## 3D volumetric imaging: benefits and applications

### General advantages

Ventricular volumetry, both on CT and MRI, has many advantages. First, when automated, it is objective and not subject to rater variance. Second, slice positioning is not impacting the three-dimensional analysis. Third, it is more sensitive to postoperative and temporal changes [15, 92]. Lastly, cohort-wide quantification, also including age-specific norms, is facilitated by automated pipelines [39, 83]. Gholampour et al. [21] evaluated linear and morphometric features to identify hydrocephalus, finding that total ventricular volume was the most important factor, followed by FOHR, underlining the superiority of VV over linear indices.

### Derived metrics

Beyond total volume, 3D analysis enables derived spatial metrics that better capture the characteristics of CSF (re)-distribution patterns of hydrocephalus. Examples are the ventricle-to-subarachnoid ratio (VSR) or quantification of convexity subarachnoid space [8, 44, 91, 100]. Anatomical subregions can, if the volumetry accounts for it, also deliver more differentiated compartment-based results, for example, comparing only changes to the temporal horns [13].

### Morphometry and vector mapping

Three-dimensional morphometric analysis of subregions has been shown to follow distinct patterns [38]. Patients with iNPH have been shown to have increased expansion of the medial aspect of the frontal horns, the superior portion of the lateral ventricles, and the temporal horns compared to age-matched controls [38].

To find changes in morphometry, the application of vector mapping is worth mentioning [1, 20, 29, 104]. When comparing two image volumes, for example, pre- and postoperative scans, a deformation field is estimated that describes how each point in one image maps to the corresponding point in the other. To identify local changes, the Jacobian matrix—comprising the first-order partial derivatives of the deformation field—quantifies how the transformation varies across space. Its determinant, the Jacobian determinant, is the key scalar quantity used in tensor-based morphometry: a value greater than 1 indicates local expansion and less than 1 indicates local compression [1]. Importantly, this analysis operates directly on the registered image volumes and does not require a segmentation mask, making it applicable to any anatomical region of interest [29].

It is defined as follows: A deformation field u(x) where y and x denote the two matrices are defined as:

$$\mathrm{u}\left(\mathrm{x}\right)= \mathrm{y} - \mathrm{x}$$

The Jacobian matrix of x = (x^1^, x^2^, x^3^) and y = (y^1^, y^2^, y^3^) is defined as follows:

$$\mathrm{J} = \left|\begin{array}{ccc} \frac{\partial {y}^{1}}{\partial {x}^{1}} & \frac{\partial {y}^{2}}{\partial {x}^{1}} & \frac{\partial {y}^{3}}{\partial {x}^{1}} \\ \frac{\partial {y}^{1}}{\partial {x}^{2}} & \frac{\partial {y}^{2}}{\partial {x}^{2}} & \frac{\partial {y}^{3}}{\partial {x}^{2}} \\ \frac{\partial {y}^{1}}{\partial {x}^{3}} & \frac{\partial {y}^{2}}{\partial {x}^{3}} & \frac{\partial {y}^{3}}{\partial {x}^{3}}\end{array}\right|$$

While the deformation of parenchyma can give indirect information about CSF-changes, the more interesting vector mapping concerns the fluid compartment itself [60, 74, 94, 96, 102]. These studies can help gain insight into the pathophysiology of CSF-flow pathologies. Their application to the differential diagnosis of ventriculomegaly remains rather limited, largely due to the lack of standardized protocols and clinically relevant cut-off values.

## Machine learning as an enabler of 3D biomarkers

### Semantic segmentation as the foundation

While early attempts at automated ventricular segmentation employed thresholding, geometric and probabilistic methods, recent segmentation algorithms use deep learning, typically a U-Net architecture [9, 10, 14, 35, 41, 67, 70, 75]. Overlap metrics, such as the Dice coefficient, routinely exceed 90%, indicating excellent performance [6, 10, 13, 14, 67].

As the configuration of the brain parenchyma can give indirect information about the configuration of the ventricular system, and uncover additional specific information, it has also enjoyed attention in hydrocephalus imaging [3, 4, 86].

Automating the segmentation process has a multitude of benefits: First, graphics processing unit (GPU)-accelerated inference surpasses manual human annotation speeds. Even when predictions are made on the central processing unit (CPU) alone, the time needed for the input is minimal. Second, automated volumetry is not subject to differences between raters, also of different levels of experience, and hence is completely reproducible. Third, analysis is easily scalable to cover population-wide cohorts. Most automated volumetry pipelines are, of course, limited to data within the same scope as the data they were trained on. When met with unseen pathologies, scanners, and population-related differences, performance is usually worse. Data augmentation strategies can leverage that to some extent by artificially expanding datasets with synthetic images. Conventionally, these were slightly altered copies of the training data, while novel approaches using diffusion models expand this to completely novel scans [89]. While augmentation strategies can partially mitigate data scarcity, they do not fully substitute for diverse, prospectively collected training data, and outcome-linked clinical validation remains limited for most available tools.

### Automated feature extraction

From these segmentations, various features can be automatically extracted. These, of course, include those mentioned in the previous chapter, yet they are usually augmented by a variety of features collectively referred to as “radiomics” [93, 108]. They can be summarized into different categories. The first category can be directly extracted from the intensity histograms and are factors such as the mean, variance, and entropy of the voxel intensities. Shape-based features are generally more relevant in hydrocephalus. They include properties such as volume, sphericity, surface area, and spherical disproportion. In contrast, the texture-based features compare local effects such as the pairwise intensities between neighbouring voxels, quantify zones of homogeneous voxel intensities, calculate the difference between a voxel and the mean of its neighbouring voxels. They can then be further multiplied by applying image filter such as wavelet or Gaussian transformations. The final spectrum of features is, of course, vast, and these describe only examples. In the context of 3D biomarkers, especially shape-based features play a vital role as variables for further analysis. While a clinician extracting the EI captures only a single dimension of a three-dimensional structure, radiomic feature extraction can quantify spatial and morphological aspects of ventricular anatomy that are either cumbersome or impossible to assess visually [56, 85, 103]. Although certain features can in principle be interpreted manually or using conventional statistical methods, their complexity, abstractness, and number necessitate dimensionality reduction before analysis. Furthermore, they can be quantified and thereby assessed objectively.

### Modelling

These aforementioned features can then be processed using classical statistics, used as input for a prediction model or clustering. Usually, the number of features is reduced to eliminate co-linearity or redundancy. Subsequently, a prediction model can be trained and validated that can have various forms: simple architectures include regressions, tree-based models, to support vector machines and neural networks for more complex data. Another approach is clustering to uncover features typical for iNPH [7]. The advances in automated segmentation and feature extraction are the prerequisites for the broad adoption of machine learning based on such features.

## Clinical impact of the 2D to 3D transition

The previously mentioned methods can be applied to a variety of clinical questions when assessing hydrocephalus. In the following chapter, the benefits of 3D volumetry to clinical problems will be addressed.

### Pediatric hydrocephalus monitoring

Since pediatric hydrocephalus patients undergo extensive follow-up periods, many studies focus on this aspect. Various studies proved the feasibility of automated volumetry in pediatric hydrocephalus on CT, MRI and ultrasound images [22, 25, 26, 28, 66, 70, 78, 88]. Subsequently, based on this volumetry, shunt failure [34], the need for CSF-diversion [63, 69], and even detection of shunt infection on imaging data has been explored [105]. Jha et al. [37] concluded that their automated volumetric segmentation approach was able to detect changes in CSF volume of symptomatic patients of scans that were considered stable by a neuroradiologist, thereby outperforming human raters. Another study by Sudevan et al. [87] showed that postoperative volumetric changes correlated strongly with clinical outcome. Other work indirectly suggests the superiority of volumetry over linear indices by framing it as the “gold standard” [64, 71]. Current models often insufficiently represent extreme cases of large ventricles, highlighting the need for models trained on heterogeneous datasets, reflecting the entire spectrum of pathological configurations in pediatric hydrocephalus [88].

### Differential diagnosis of ventriculomegaly

Unlike pediatric hydrocephalus, ventriculomegaly in adults has different implications for diagnosis and treatment. Its differential diagnosis in the context of cognitive decline has been extensively studied, particularly in distinguishing iNPH from Alzheimer's disease (AD) and progressive supranuclear palsy (PSP) [3, 12, 21, 23, 58, 84, 100, 103, 106]. Volumetric and radiomic approaches are well suited to this problem because the underlying pathologies produce distinct spatial signatures—differences in compartment-specific volumes, CSF redistribution patterns, and tissue texture—that linear indices cannot capture with equal precision. 3D subarachnoid space volumetry can quantify the DESH pattern directly [100] while morphometric and radiomic features have shown to uncover subtle differences such as local ventricular shape and white matter texture across groups [3].

Applied through conventional statistics, volumetric measures already outperform linear indices in relation to diagnostic accuracy and relate more strongly to gait and cognition [12, 44]. Machine learning raises performance further [4, 7, 50, 56, 58, 68, 77, 103]. Methodology, compared cohorts, and the extracted features are heterogeneous [19]. Most use a supervised learning technique in the form of a regression or support vector machine (SVM). Most of them have excellent results, reporting AUCs exceeding 0.9 [4, 50, 56, 77, 103, 106]. Especially for the differentiation of PSP from iNPH results of AUCs of 0.95 −1.0 are reported [4, 77]. Also, clustering approaches are able to differentiate iNPH from healthy controls, where, interestingly, also the CSF regions reflecting DESH were the most important regions for identifying iNPH [7]. Inspired by this, attempts were made to quantify DESH [2, 101]. Furthermore, two new indices were introduced: The total ventricular volume divided by the high convexity subarachnoid space (SAS), named “Venthi index”, and the “Sylhi index” as the volume of the Sylvian fissure and basal cistern divided by high convexity SAS [101]. While qDESH was validated and showed excellent diagnostic results, both novel indices still need validation and definitions of cut-off values.

Other studies also compared region-specific parenchymal volumes for the diagnosis of iNPH [3, 27, 31, 48, 58, 76, 80, 95]. While changes to the ventricular morphology far surpass changes to the parenchyma for the differentiation of iNPH and AD to healthy controls, grey matter volume seems preserved in iNPH patients compared to healthy controls, while the white matter appears reduced [3, 27, 76].

### Preoperative prediction of response to CSF-diversion

Commonly used diagnostic tools for evaluating the indication for CSF-diversion procedures have limited diagnostic odds ratios or are invasive [90]. Hence, improving the non-invasive image-based parameters is of considerable clinical value [54, 84, 97]. Different methods, input features, and cohort sizes have been applied to this problem [6, 19, 24, 49, 52, 57, 72, 85, 97]. A prediction model in the form of a support vector machine (SVM) is frequently used, and a performance increase when clinical data is augmented with imaging seems notable [19, 62].

Thavarajasingam et al. [90] found in a meta-analysis that the only predictors of shunt response were CA and changes to the periventricular white matter. Yet, the EI and DESH were not, despite their inclusion in guidelines [59]. With the adjunct of machine learning, excellent identification of responders to shunt surgery seems possible [19, 33, 97]. Also in this task, excellent AUC values of up to 0.94 were achieved for predicting response to CSF-drainage in probable iNPH patients, although only considering the tap-test and not definitive shunt placement [97]. Not only the ventricular volume but also regions of the parenchyma were of high importance in feature analysis [80, 97]: Large volumes of the inferior lateral ventricle, hippocampus, certain fronto-orbital gyri, and small posterior and parietal white matter were distinct features of patients who responded well to CSF-diversion [97].

### Post-shunt management and longitudinal follow-up

After CSF-diversion surgery, changes of VV can happen subtly and over long periods of time [11, 28, 42]. When fluctuations in symptoms occur, it can sometimes be difficult to correlate this with imaging and draw clinical conclusions. The quantification of perioperative changes in VV is possible, and VV has been shown to be more sensitive to change than the EI [53, 61]. Shunt malfunction in adults can also be detected with high sensitivity using volumetry based models [32, 40, 82]. The need for shunt revision surgery was correctly identified in 92% in a study by Huang et al. [32], general shuntmal function by Kellogg et al. [40] with an AUC of 0.933, and ventricular volume reduction after shunt surgery by Holmgren et al. [30].

## Practical barriers and implementation challenges

As shown in the previous sections, 3D biomarkers have many applications and are often superior to 2D ones in hydrocephalus. Yet, their clinical application is very limited. Numerous factors can be attributed to that. First, many models are not accompanied by a ready-to-use inference software. They need coding knowledge to be applied. Second, GPU for rapid inference is still scarce in hospitals. Third, regulatory and quality-control requirements need to be met, too. Commercial applications of ready-to-use models in clinics are generally tied to financial constraints. A possible solution to many of these obstacles could be the integration of academic, center-tailored models that are integrated into the radiological workflow [43]. Commercially validated products exist for volumetry, e.g. cNeuro (Combinostics, Tampere, Finland), and they are used for further analysis [47, 55, 79, 107]. However, when confronted with distortive factors such as postoperative changes, e.g., shunt artifacts, many models that are not trained on this specific data tend to show inferior performance. Models trained on large, heterogeneous datasets that reflect the spectrum of configurations and pathologies of the target inference population are warranted.

## Research gaps and future direction

The approaches described achieve strong metric performance, yet clinical translation remains limited. Most models were trained and validated on small, often single-center datasets, and external validation across diverse cohorts, scanners, and pathologies is largely absent. Reports of AUCs of 1.0 [77] for the iNPH/PSP differentiation on a dataset of 53/65 cases invite external validation, as acknowledged by the authors, to ensure no overfitting occurred. Equally, clinically meaningful thresholds for volumetric change—the minimum difference that should prompt a clinical decision – have not been defined consistently. Standardized reporting frameworks for 3D biomarkers in hydrocephalus that are integrated into guidelines are still lacking. Prospective studies comparing outcomes of novel assessment strategies using 3D biomarkers to manual inspection are lacking, too. Deep learning models are often described as “black boxes”, meaning that how they arrive at a decision cannot always be understood. To mitigate this, attempts made at explaining how AI-algorithms work, yet their application is scarce [18]. Addressing these gaps would lay the groundwork for longitudinal biomarker trajectories to be integrated reliably into clinical workflows, and ultimately into multimodal predictive models that could inform surgical and medical decision-making.

**Fig. 2:**
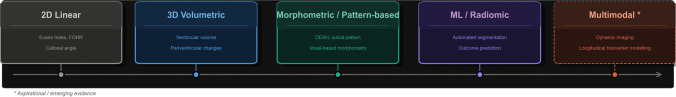
Schematic illustration of the progression of radiological assessment of hydrocephalus

While many models incorporate partial aspects, a gap is the combination of features of ventricular morphology, changes to the parenchyma, and maybe even combined with MR protocols that target CSF dynamics [98]. The evolution of hydrocephalus imaging from past, to present and to possible futures is illustrated in Fig. 2. Also, future models should clearly differentiate between diagnosis, monitoring, and supporting preoperative decision-making. As the imaging modality can also differ, e.g., suspected shunt malfunction is rarely assessed on MRI, multimodal input accounting for the limited availability of optimal data, taking the best available data should be pursued.

## Conclusion

Linear indices are still the backbone of routine hydrocephalus assessment, but they have inferior performance compared to 3D biomarkers in precise diagnosis, treatment monitoring, and longitudinal follow-up. Volumetric, morphometric, and radiomic features address many of these shortcomings, and machine learning has made their application at scale realistic. Factors still missing are external validation, agreed thresholds for clinically meaningful change, incorporation into radiological workflows, open-access publishing, and embedding these tools into guidelines. Ultimately, the goal should be to prove improved patient care, a level of evidence that has not yet been achieved.

## Data Availability

No datasets were generated or analysed during the current study.

## Abbreviations

AUC: Area under the Curve
BVR: Brain per Ventricle Ratio
CA: Callosal Angle
CPU: Central Processing Unit
CSF: Cerebrospinal Fluid
DESH: Disproportionately Enlarged Subarachnoid Space Hydrocephalus
EI: Evans Index
FOHR: Frontal and Occipital Horn Ratio
GPU: Graphics Processing Unit
iNPH: Idiopathic Normal Pressure Hydrocephalus
PSP: Progressive Supranuclear Palsy
SVM: Support Vector Machine
SAS: Subarachnoid Space
VBR: Ventricle to Brain Ratio
VV: Ventricular Volume
VSR: Ventricle to Subarachnoid Volume Ratio
